# Early corneal nerve fibre damage and increased Langerhans cell density in children with type 1 diabetes mellitus

**DOI:** 10.1038/s41598-019-45116-z

**Published:** 2019-06-19

**Authors:** Maryam Ferdousi, Kenneth Romanchuk, Jean K. Mah, Heidi Virtanen, Christine Millar, Rayaz A. Malik, Danièle Pacaud

**Affiliations:** 1 Division of Cardiovascular Sciences, University of Manchester and NIHR/Wellcome Trust Clinical Research Facility, Manchester, M13 9NT UK; 2 University of Calgary, Calgary, AB Canada; 3 Alberta Children’s Hospital, Calgary, AB Canada; 4 Alberta Children’s Hospital Research Institute, Calgary, AB Canada; 5 Weill-Cornell Medicine-Qatar, Doha, Qatar

**Keywords:** Diabetes complications, Paediatric research

## Abstract

Corneal confocal microscopy (CCM) has been used to identify corneal nerve damage and increased Langerhans cell (LC) density in adults with Type 1 diabetes mellitus (T1DM). The purpose of this study was to evaluate whether corneal confocal microscopy can identify early corneal nerve damage and change in LC density in children and adolescents with T1DM. 64 participants with T1DM (age-14.6 ± 2.5 years, duration of diabetes-9.1 ± 2.7 years, HbA1c-75.66 ± 2.53 mmol/mol [9.1 ± 1.8%]) and 48 age-matched healthy control subjects underwent CCM. Sub-basal corneal nerve morphology and the density of mature and immature LCs was quantified. Corneal nerve fibre length and branch density were lower, whilst fibre density and tortuosity did not differ and both immature and mature LC density was significantly higher in T1DM compared to control subjects. There was no association between HbA1c and duration of diabetes with nerve fibre parameters or LC’s density. Children and adolescents with T1DM demonstrate early immune activation and nerve degeneration.

## Introduction

Advanced diabetic neuropathy is a cause of significant morbidity and mortality. It is therefore important to identify early nerve damage to limit the development and progression of this complication. We and others have demonstrated the utility of corneal confocal microscopy in identifying early corneal nerve loss in adults and small cohorts of young adolescents with Type 1 diabetes mellitus (T1DM) without neuropathy^1–5^ and was not associated with dry eye^6^. We have also demonstrated corneal nerve loss in adults and children with T1DM without retinopathy and microalbuminuria, suggesting that this may be the earliest microvascular complication^7,8^. Of prognostic relevance, a reduction in corneal nerve fibre length has been shown to predict the development of diabetic neuropathy in adults with T1DM^9,10^.

Corneal Langerhans cells (LC’s), first identified by Engelmann in 1867^11^, are bone marrow derived antigen-presenting cells and a component of the corneal immune defence system^12^, located throughout the corneal epithelium^13^. LC’s in the central cornea are immature cells without dendritic structures while those in the periphery are mature and possess dendritic structures^14^. They reside primarily in the basal epithelium or sub-basal layer^15^ and several studies have used corneal confocal microscopy (CCM) to quantify LC’s in normal and pathologic human corneas^11,15,16^.

Studies have reported a link between LC’s and neuropathy with an increase in epidermal LC density being related to a reduction in the intra epidermal nerve fibre density (IENFD) in the footpad of Streptozotocin (STZ) diabetic rats^17^. Epidermal LC density has also been shown to be increased and related to reduced IENFD in patients with painful diabetic neuropathy^18^. Both epidermal and sub-epidermal LC’s are increased in the db/db mouse in the initial phase of mechanical allodynia and then decrease when mechanical allodynia diminishes^19^. We have previously demonstrated a three-fold increase in the density of LC’s in the central cornea of patients with diabetes with no or mild neuropathy^20^. However, in that study we were using a first-generation white light corneal confocal microscope, which cannot differentiate mature from immature LC’s. We have undertaken corneal confocal microscopy to quantify sub-basal corneal nerve pathology and LCs density in a large cohort of children and adolescents with T1DM. This may allow us to assess the utility of CCM in identifying early nerve fibre damage and insights into the mechanisms underlying early corneal nerve fibre loss in children with T1DM.

## Research Design and Methods

### Study subjects

This study was approved by the Conjoint Health Research Ethics Board at the University of Calgary and Alberta Children’s Hospital. Children with a history of at least 5 years of T1DM aged 8 to 18 years and healthy age-matched control subjects were invited to participate. Subjects were recruited through the Alberta Children’s Hospital Diabetes Clinic and healthy controls were recruited from the patient’s family, relatives and through advertisement posters in general paediatric or paediatric ophthalmology clinics. Subjects with a known history of corneal abnormality, trauma or surgery, wearing contact lenses, any other cause of neuropathy, uncontrolled hypothyroidism and celiac disease were excluded from the study. This study adhered to the tenets of the Declaration of Helsinki and informed written consent was obtained from all participants aged 18 years old and from a parent and/or legal guardian of participants aged less than 18 years old.

### Corneal confocal microscopy

All the participants underwent CCM using the Heidelberg Retinal Tomograph III Rostock Cornea Module (Heidelberg Engineering GmbH, Heidelberg, Germany) according to our previously published procedures^21^. HRT III is a class 1-laser system with 400 × 400 μm field of view and 10 μm/pixel optical resolution with a 2D digital image size of 384 × 384 pixels. Images were captured from the centre of the cornea using the section mode. Patients were assessed and CCM was performed at the Alberta Children’s hospital by three well-trained examiners and we have previously demonstrated the feasibility and reproducibility of this technique in children^22,23^. All participants were examined by an ophthalmologist after the CCM was performed to assess for any corneal alterations.

Demographic data included; age, gender, duration of diabetes, self-assessment pubertal stage (tanner stage I–V)^24^ and HbA1c. Six CCM images (3 per eye) of the corneal sub-basal nerve plexus were selected from 10–18 images and were analysed by a single examiner in a masked manner^25^. All the images were analysed manually using purpose-designed software (CCMetrics, MA Dabbah; Imaging Science and Biomedical Engineering, University of Manchester, Manchester, UK)^26^. Corneal nerves were traced using a digital pen and a wide screen tablet (Trust international B.V., China). Corneal nerve fibre morphological parameters included corneal nerve fibre density (CNFD); a measure of the total number of main corneal nerves/mm^2^, corneal nerve branch density (CNBD); the number of junctions between branches and main nerves/mm^2^, corneal nerve fibre length (CNFL); the total length of all corneal nerve fibres (mm/mm^2^) and corneal nerve fibre tortuosity (CNFT), as expressed by the tortuosity coefficient (TC).

LC’s were defined as bright dendritic structures in the images used to analyse the sub-basal corneal nerves^11^. LC’s less than 50 µm in length without dendritic structures were considered immature cells and those with a length greater than 50 µm and dendritic structures were considered as mature cells. The LC’s density (no./mm^2^) was derived by counting the total number of cells in the area of the cornea using the NBD feature and the length of each cell was calculated using the NFL feature of CCMetrics.

### Statistical analysis

IBM SPSS v22 (Chicago, IL, USA) for Windows was used to analyse the results. Data are expressed as Mean ± SD and were tested for normality. Two independent sample t tests (for parametric variables) and Mann-Whitney U test (for non-parametric variable) were used to compare means between the two groups. Pearson correlation coefficient (Spearman rank correlation coefficient for non-parametric data) was used to assess the correlation between corneal LCs’ density and other clinical data. To assess categorical data, Pearson’s Chi-square (χ^2^) test of independence and a Fisher’s Exact test were used.

## Results

Sixty-four subjects with T1DM (age: 14.6 ± 2.5 years, duration diabetes: 9.1 ± 2.7 years and HbA1c: 75.66 ± 2.53 mmol/mol [9.1 ± 1.8%]) and 55 aged-matched healthy controls (age: 13.6 ± 3.1 years) were studied. The demographic and CCM results of these subjects are summarized in Table 1.

**Table 1:** Demographic and corneal confocal microscopy findings in children with Type 1 diabetes mellitus and age matched healthy controls.

Parameters	T1DM	Controls	P value
Number	64	55	NA
Age	14.6 ± 2.5	13.6 ± 3.1	0.06
Gender (female %)	48.4%	60.8%	0.1
Duration of diabetes (years)	9.1 ± 2.7	NA	NA
HbA1c (mmol/mol) [%]	75.66 ± 2.53 [9.1 ± 1.8]	NA	NA
Corneal nerve fibre density (no./mm^2^)	31.4 ± 7.6	31.5 ± 6.8	0.9
Corneal nerve branch density (no/mm^2^)	72.3 ± 29.4	85.7 ± 36.8	0.03
Corneal nerve fibre length (mm/mm^2^)	22.8 ± 4.9	24.8 ± 5.9	0.04
Corneal nerve fibre tortuosity (TC)	13.8 ± 5.2	12.9 ± 2.9	0.2
Participants with LC’s (%)	85.9%	69.1%	0.04
Mature LC density (no./mm^2^)	2.7 ± 4.3	1.3 ± 2.09	0.04
Immature LC density (no./mm^2^)	48.9 ± 65.5	16.6 ± 21.6	0.005
Total density of LC’s (no./mm^2^)	51.6 ± 68.6	17.9 ± 22.6	0.005

All data are presented as Mean ± SD.

### Corneal nerve morphology

**Figure 1:**
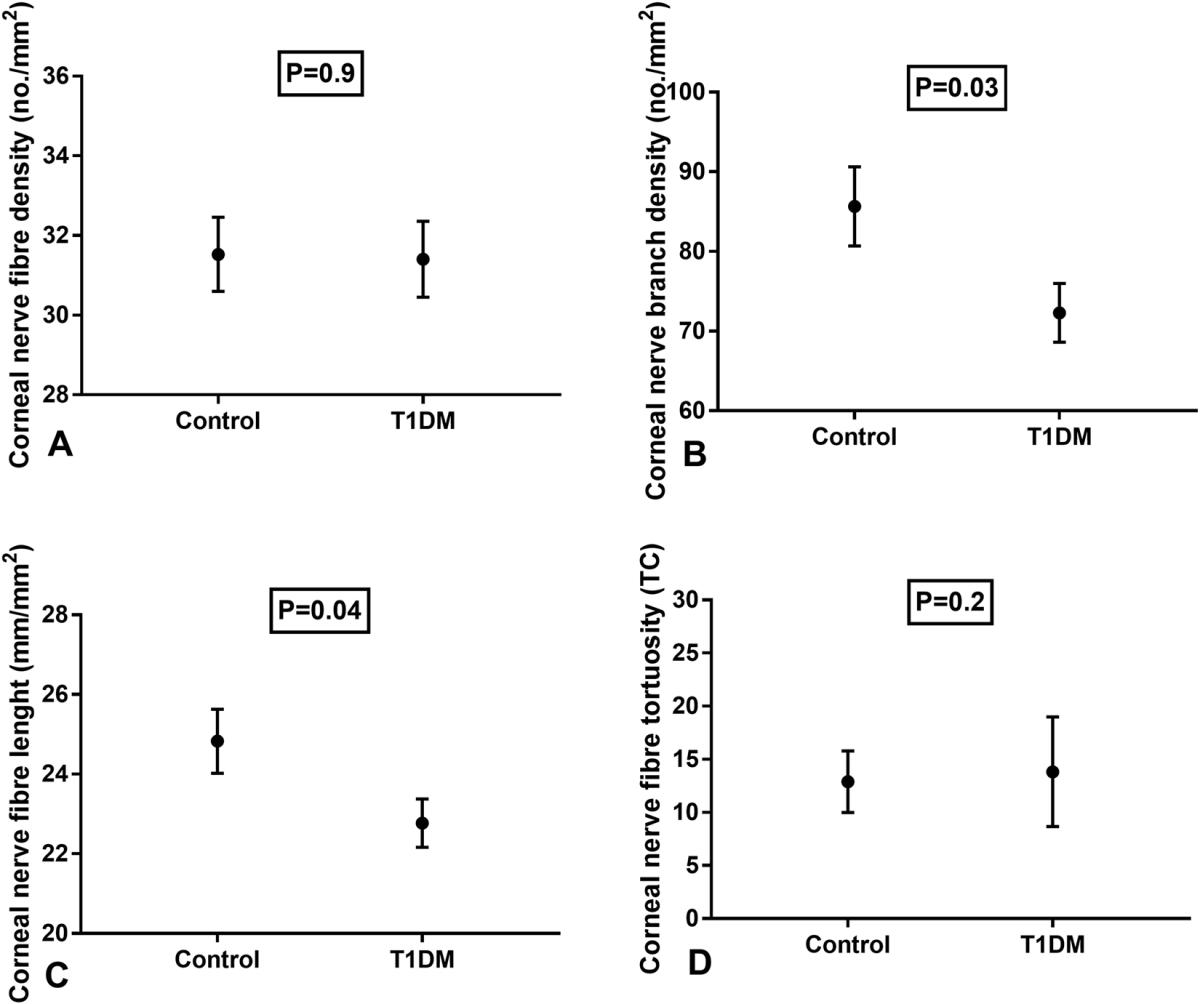
Corneal nerve fibre parameters in children with Type 1 diabetes mellitus and healthy controls (bars indicate one standard error). (**A**) Corneal nerve fibre density (no./mm^2^). (**B**) Corneal nerve fibre branch density (no/mm^2^). (**C**) Corneal nerve fibre length (mm/mm^2^). (**D**) Corneal nerve fibre tortuosity (TC).

CNFL (mm/mm^2^) (22.8 ± 4.9 vs 24.8 ± 5.9, P = 0.03) and CNBD (no./mm^2^) (72.3 ± 29.4 vs 85.7 ± 36.8, P = 0.04) were significantly lower, with no difference in CNFD (no./mm^2^) (31.4 ± 7.6 vs 31.5 ± 6.8, P = 0.9) and CNFT (TC) (13.8 ± 5.2 vs 12.9 ± 2.9, P = 0.2) between individuals with T1DM and healthy controls (Fig. 1). No significant correlation was found between corneal nerve parameters with age, HbA1c and duration of diabetes.

### Langerhans cells

**Figure 2:**
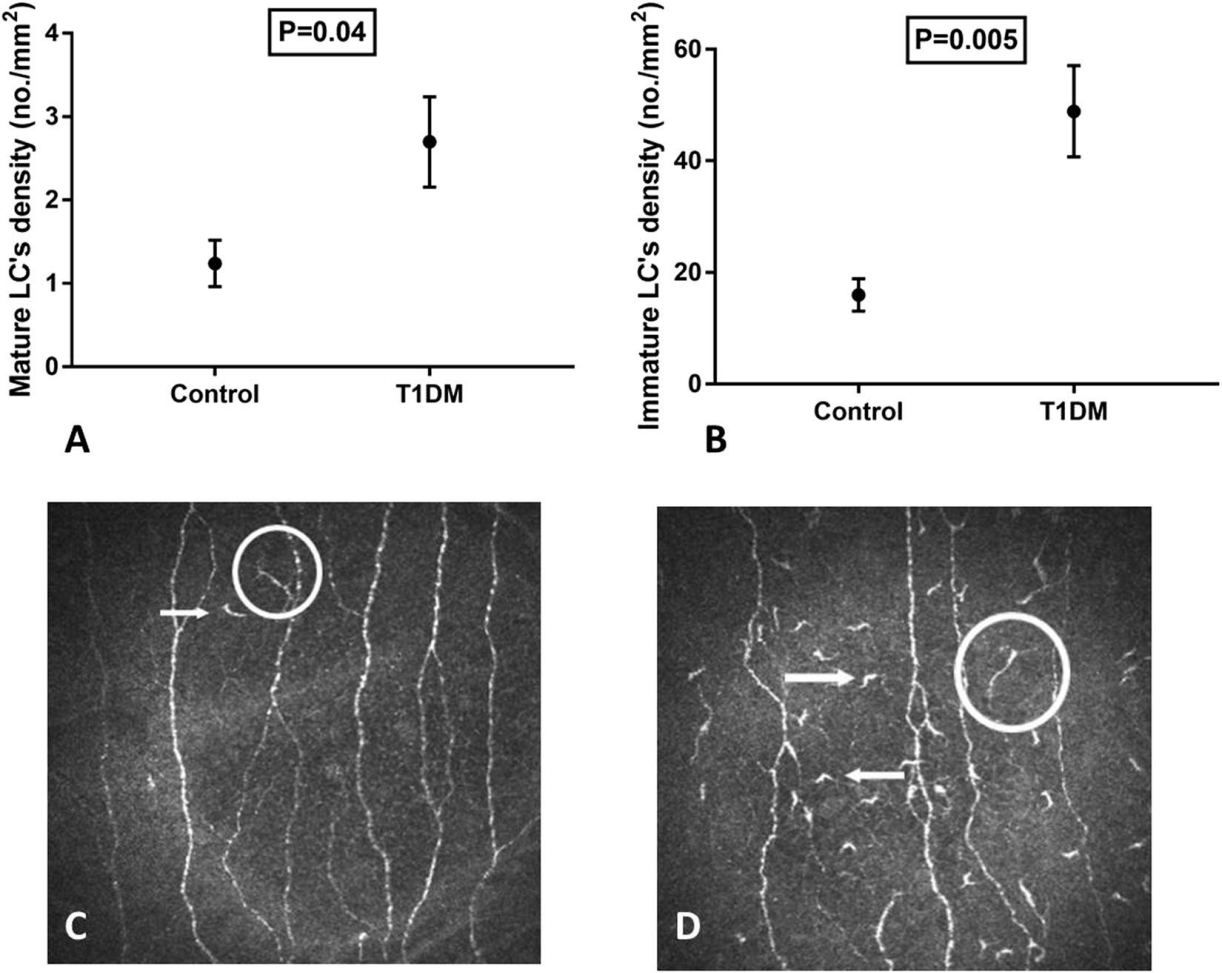
(**A**) Mature LC’s density (no./mm^2^) in children with Type 1 diabetes mellitus and healthy controls (bars indicate one standard error). (**B**) Immature LC’s density (no./mm^2^) in children with Type 1 diabetes mellitus and healthy controls (bars indicate one standard error). (**C**) A CCM image of the corneal sub-basal nerve plexus in a healthy control. (**D**) A CCM image in a child with Type 1 diabetes mellitus showing a reduction in corneal nerves and increased immature (arrows) and mature (circle) LC’s.

Mature LC density (no./mm^2^) (2.7 ± 4.3 vs 1.3 ± 2.09, P = 0.04), immature LC density (no./mm^2^) (48.9 ± 65.5 vs 16.6 ± 21.6, P = 0.005) and the total density of LC’s (no./mm^2^) (51.6 ± 68.6 vs 17.9 ± 22.6, P = 0.005) were significantly higher in individuals with T1DM compared to healthy controls (Fig. 2). The proportion of individuals with immature (85.9% vs 69.01%, P = 0.04) and mature (60% vs 38.2%, P = 0.04) LC’s was significantly greater in patients with T1DM compared to control subjects.

### Correlations

CNFD (r = 0.2, P = 0.01), age (r = 0.2, P = 0.03) and pubertal stage (Tanner staging) (r = 0.34, P = 0.005) correlated significantly with the density of mature LC’s in patients with T1DM. Pubertal stage correlated significantly with the total LC density (r = 0.25, P = 0.04) and immature LC density (r = 0.2, P = 0.02) in patients with T1DM, but not in controls. The proportion of participants who had gone through puberty did not differ significantly between control subjects (44/55–80%) and participants with T1DM (59/64–90%). The duration of diabetes, HbA1c, CNBD, CNFL and CNFT did not correlate with LC density.

## Discussion

Subclinical DN has been reported in 25% of newly diagnosed and 50% of children within 5 years of being diagnosed with T1DM^27^. However, DN is often asymptomatic in children and early diagnosis is difficult. Techniques used to detect early DN have limited utility in children because they are subjective (symptoms/signs, quantitative sensory testing), uncomfortable (nerve conduction studies) or invasive (skin biopsies)^27^. We have established CCM as an objective measure of DN in adults with T1DM, T2DM and subjects with impaired glucose tolerance^2,28–30^.

In a pilot study, we previously reported that CCM is an acceptable procedure, but we found no abnormality of corneal nerves in a small cohort of children with T1DM or T2DM^23^. However, we and others have recently shown that there is an early abnormality in corneal nerve fibres in larger cohorts of children and adolescents with T1DM^4,8^. Certainly, studies in adults with diabetes show a global reduction in CNFD, CNBD and CNFL and an increase in CNFT^31^. We now show a significant reduction in corneal nerve branch density and length with preserved nerve fibre density and tortuosity in a large cohort of children with T1DM. This reduction in CNBD and CNFL represents the earliest pathology to the most distal nerves, sparing the more proximal major nerves represented by CNFD. Indeed, an increase in CNBD is indicative of nerve fibre regeneration in individuals treated with CSII^32^ and after simultaneous pancreas and kidney transplantation^29^. An increase in corneal nerve fibre area has been shown to be the earliest measure of nerve fibre repair^33^. Indeed, we have also shown that more prominent nerve fibre loss at the inferior whorl reflects the earliest damage to the most distal nerve fibres, consistent with the dying back process in diabetic neuropathy^34^.

Previous *ex vivo*^35^ and *in vivo*^20^ studies have shown an increase in LC density in the diabetic cornea. We show a threefold increase in the density of both mature and immature LC’s in the diabetic cornea. Studies have suggested a possible interaction between LC’s and nerves in the pathogenesis of neuropathy^36^, indicating neuro-immune communication^37^. Nerves can also influence immune cell activity by releasing cytokines and neuropeptides^38^, with relevant neuropeptide receptors being expressed by resident immune cells^39^. DC’s express neurotrophic factors such as CNTF, which promotes re-innervation and corneal denervation has been associated with a reduction in DC density and altered morphology^40^. Leppin *et al*.^35^ demonstrated a significant increase in DC’s using CCM and related it to total corneal nerve fibre length in STZ-diabetic mice. We now confirm and extend our previous observations showing a significant increase in the density of LC’s in patients with diabetes but find no correlation with the severity of corneal nerve damage, HbA1c or duration of diabetes. There was a correlation between pubertal stage and the density of LC’s in T1DM, but not in controls even though the proportion of participants who had gone through puberty was comparable. This requires further study as there is a link between pubertal stage and progression of neuropathy in youth with diabetes^41^. Ideally this requires a longitudinal study to assess the temporal change in LC density and corneal nerve morphology during puberty to see if this differs between patients with T1DM and controls.

In conclusion, CCM has identified a small but significant reduction in corneal nerve branch density and length and an increase in LC’s in children and adolescents with T1DM. This early corneal nerve damage was not related to glycemic control, diabetes duration or LC density. The role of CCM as an early diagnostic marker of sub-clinical diabetic neuropathy in children and the potential role of LC’s in mediating this early pathology merits further study.

## Data Availability

The dataset generated during and/or analysed during the current study are available from the corresponding author on reasonable request.
